# Primary Spinal Intradural Hydatid Cysts in a Child With Progressive Paraparesis: A Case Report and Literature Review

**DOI:** 10.1002/ccr3.71823

**Published:** 2026-01-08

**Authors:** Afshar Abolhasani, Abdolnaser Farzan, Amirreza Keyvanfar, Mahtab Khodabandelu

**Affiliations:** ^1^ Department of Neurosurgery, School of Medicine Shahid Beheshti University of Medical Sciences Tehran Iran; ^2^ Department of Neurosurgery, Educational and Therapeutic Center, Mofid Children's Hospital Shahid Beheshti University of Medical Sciences Tehran Iran; ^3^ Infectious Diseases and Tropical Medicine Research Center Shahid Beheshti University of Medical Sciences Tehran Iran; ^4^ Department of Pharmacy, School of Pharmacy Zanjan University of Medical Sciences Zanjan Iran

**Keywords:** child, echinococcosis, *Echinococcus granulosus*, hydatid cyst, neurosurgery, paraparesis

## Abstract

Primary spinal hydatid cyst is an uncommon form of echinococcosis, which may cause focal neurological deficits. We report an extremely rare case of spinal hydatic cyst with several diagnostic and therapeutic challenges. A 4‐year‐old boy was referred to our medical center suffering from progressive lower back pain, weakness and numbness in both lower limbs, and urinary incontinence for 4 months. He was initially misdiagnosed with rheumatoid arthritis (RA). However, as his clinical manifestations deteriorated, a neurosurgical consultation was requested to evaluate other differential diagnoses. Neurological examination revealed bilateral lower extremity weakness accompanied by increased deep tendon reflex (DTR) of the lower extremities. Thoracolumbar magnetic resonance imaging (MRI) demonstrated multicystic lesions, extramedullary intradural, extending from T8 to S2, which caused compression and posterior displacement of the cord. The patient underwent surgery and medical treatment with albendazole and praziquantel. Although early postoperative imaging showed no residual cysts, he experienced recurrence on follow‐up. Due to the nonspecific manifestations and high morbidity of spinal hydatidosis, a multidisciplinary approach should be considered to diagnose and manage it. If spinal hydatid disease is diagnosed promptly, it can preclude severe complications, such as cyst expansion and subsequent spinal cord damage.

## Key Clinical Message

For pediatric spinal cord compression in endemic areas, suspect hydatid cysts. Timely diagnosis, rupture‐free surgical resection, and long‐term follow‐up are vital to mitigate neurological injury and high recurrence rates.

## 1 Introduction

Hydatid disease is a zoonotic infection caused by *Echinococcus* tapeworm. Although dogs, cats, and livestock are hosts of this parasite [1], humans may become infected through consumption of contaminated food or water. *Echinococcus* species can involve the liver and lungs of the host in the larval stage [2]. Among the different *Echinococcus* species, *Echinococcus granulosus* and *E. multilocularis* most commonly affect humans [3].

Rural and nomadic communities in endemic countries like China, India, Peru, and parts of Africa and the Middle East are mainly affected by this tropical disease. Hydatid disease imposes a substantial disease burden on these regions [4, 5]. At the national level, a meta‐analysis by Mahmoudi et al. [6] revealed that the prevalence of hydatid cysts was significantly higher in young villagers and nomads in the North and West of Iran.

Hydatid disease of the bone is extremely rare, occurring in only 0.5%–2% of the involvements [1]. Approximately half of the cases of bone hydatidosis involve the spinal cord [3]. Additionally, cystic echinococcosis of the central nervous system (CNS) accounts for up to 3% of hydatid cyst cases reported worldwide [7]. Spinal hydatidosis is extremely rare in pediatrics and adolescents [7, 8]. This situation can be attributed to underreporting of the disease, particularly in endemic regions. Spinal hydatid disease is categorized by Braithwaite et al. [9] into five classes based on the location of involvement: intramedullary (type I), intradural and extramedullary (type II), extradural and intraspinal (type III), vertebral body (type IV), and paravertebral (type V). In addition, hydatid disease of the spine is classified as primary or secondary based on the route of infection [10].

Spinal hydatidosis primarily occurs extradurally, presenting with multiple cysts. Intradural extramedullary cysts are extremely rare. Typically, spinal involvement arises from direct extension of the disease from the abdominopelvic cavity or thorax. The dorsal area of the spinal cord is mainly involved in the thoracic, lumbar, cervical, and sacral regions [11, 12, 13, 14].

In this article, we report a case of primary spinal intradural extramedullary hydatid cysts in a child with progressive paraparesis, who experienced recurrence after appropriate surgical and medical treatment. The diagnosis and treatment of our case were interesting and challenging, so sharing it can be helpful for clinicians. CNS echinococcosis is a life‐threatening infection that requires a multidisciplinary approach for its timely diagnosis and management.

## 2 Case History and Examinations

A 4‐year‐old boy was referred to the Mofid Children's Hospital (Tehran, Iran) suffering from lower back pain, weakness and numbness in both lower limbs, and urinary incontinence. His symptoms emerged 4 months ago and progressed over time. He was initially evaluated for RA and treated with corticosteroids. However, as his clinical manifestations deteriorated, a neurosurgical consultation was requested to evaluate other differential diagnoses.

Upon admission, his vital signs were as follows: oral body temperature = 37.4°C, blood pressure = 108/64 mmHg, pulse rate = 110 beats/min, respiratory rate = 20 beats/min. Neurological examination revealed bilateral weakness of the lower extremities (muscle force: 3/5). DTR of the lower extremities was hyper. No sensory level found in the examination. In addition, no visible deformity and no sign of external wound or abscess on his back was seen.

## 3 Investigations

The results of laboratory assessments were nonspecific: white blood cell count = 8900 cells/mm^3^ (48% neutrophil, 32% lymphocyte, 5% monocyte, and 18% eosinophil), hemoglobin = 12.5 mg/dL, platelet count = 235,000 cells/mm^3^, erythrocyte sedimentation rate (ESR) = 20 mm/h, C‐reactive protein (CRP) = 0.25 mg/dL, aspartate aminotransferase (AST) = 18 U/L, alanine transaminase (ALT) = 12 U/L, alkaline phosphatase (ALP) = 102 U/L, Direct Bilirubin = 0.2 mg/dL, and Total Bilirubin = 0.8 mg/dL. Using the enzyme‐linked immunosorbent assay (ELISA), serologic IgG tested positive for Echinococcosis.

**FIGURE 1:**
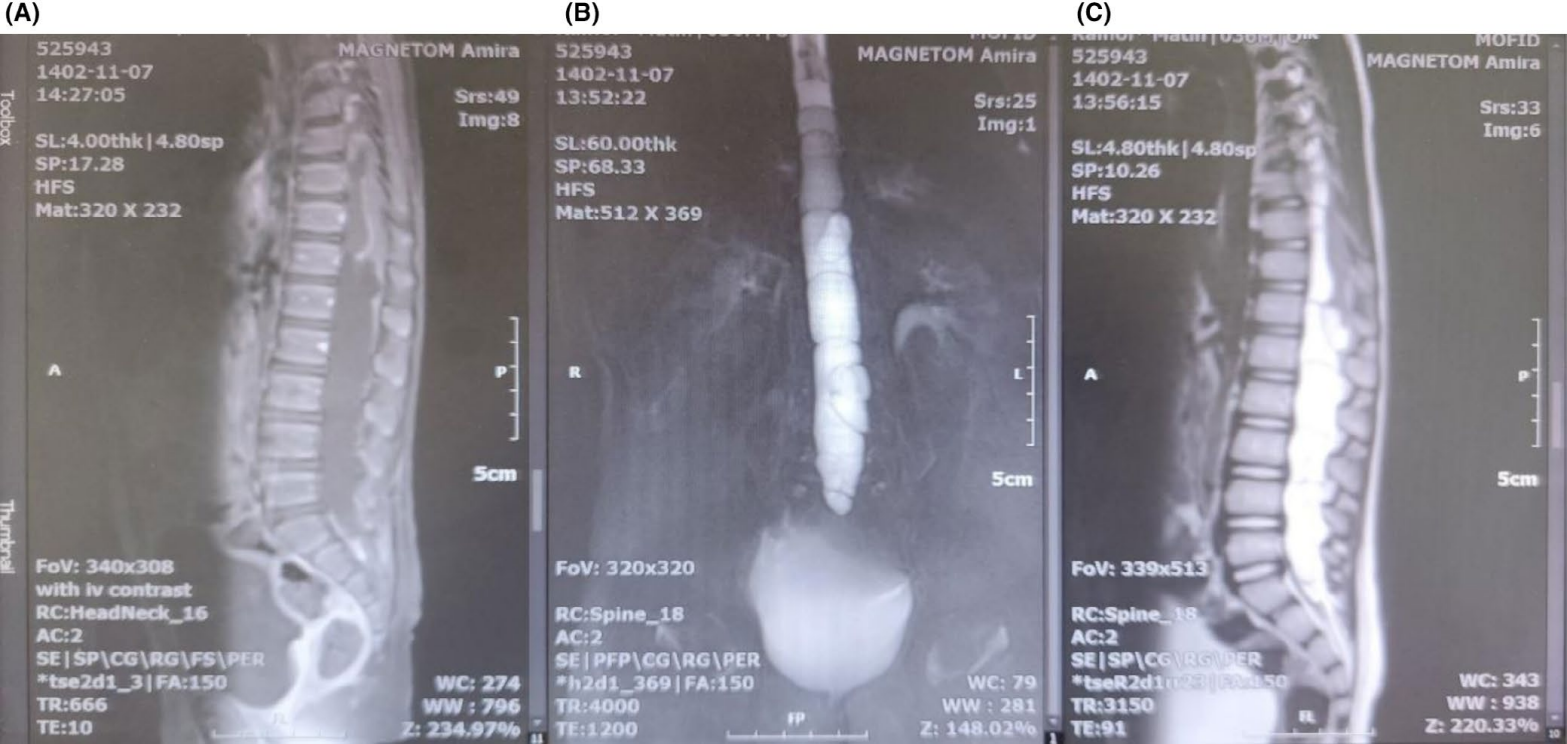
Preoperative thoracolumbar imaging. (A) T1‐weighted MRI with gadolinium injection (left), (B) Myelogram (middle), (C) T2‐weighted MRI with gadolinium injection (right). They depict well‐defined large hetrosignal multicystic lesions extramedullary intradural extending from T8 to S2 that caused compression and posterior displacement of cord.

Further investigation on thoracolumbar MRI demonstrated well‐defined, large heterosignal multicystic lesions extramedullary intradural extending from T8 to S2 that caused compression and posterior displacement of the cord (Figure 1). Thoracoabdominal computed tomography (CT) scan confirmed there was no lesion in other organs, including liver, lungs, and kidneys.

## 4 Treatment

**FIGURE 2:**
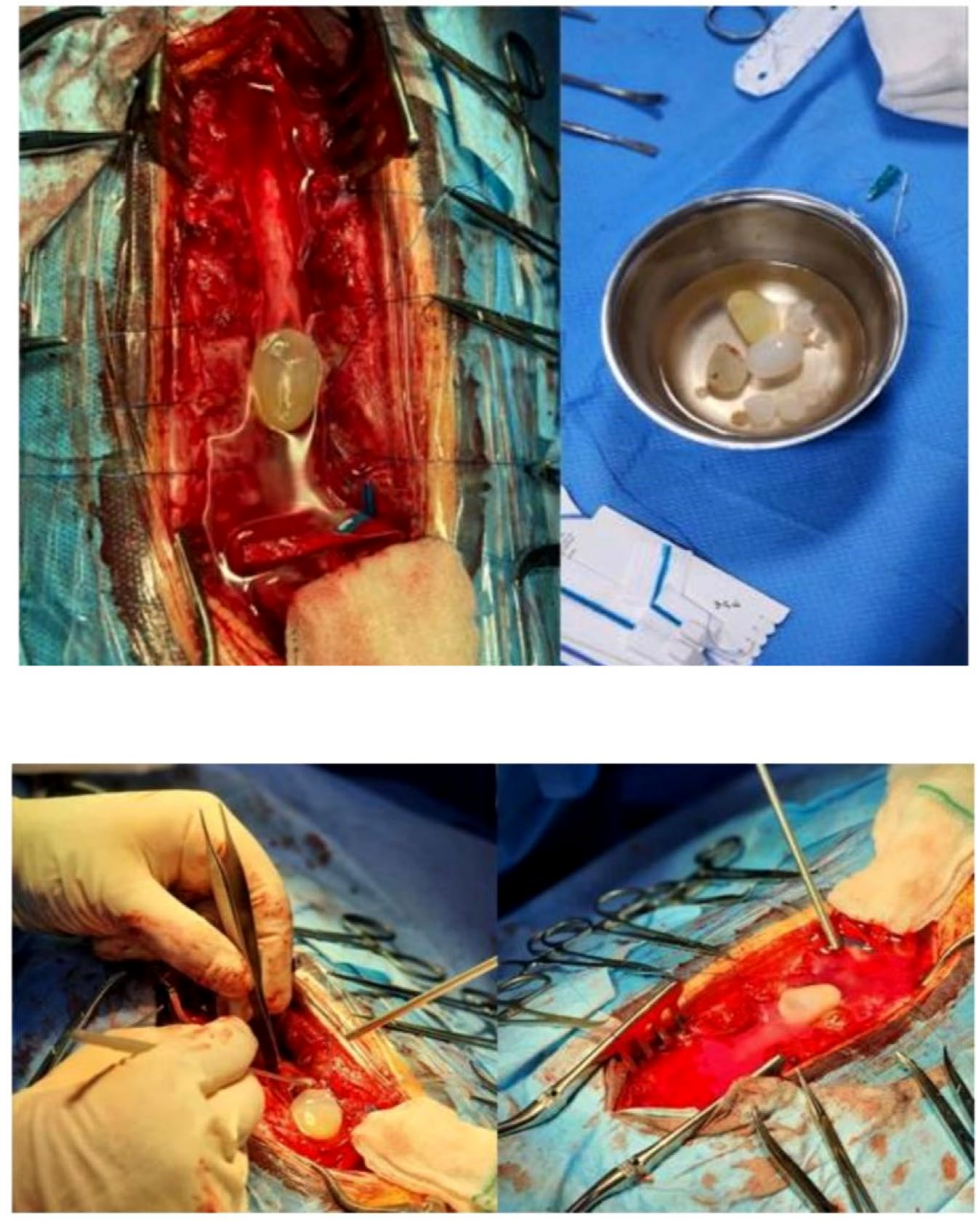
Intraoperative imaging. Multiple cysts that were totally located in the intradural extramedullary space.

**FIGURE 3:**
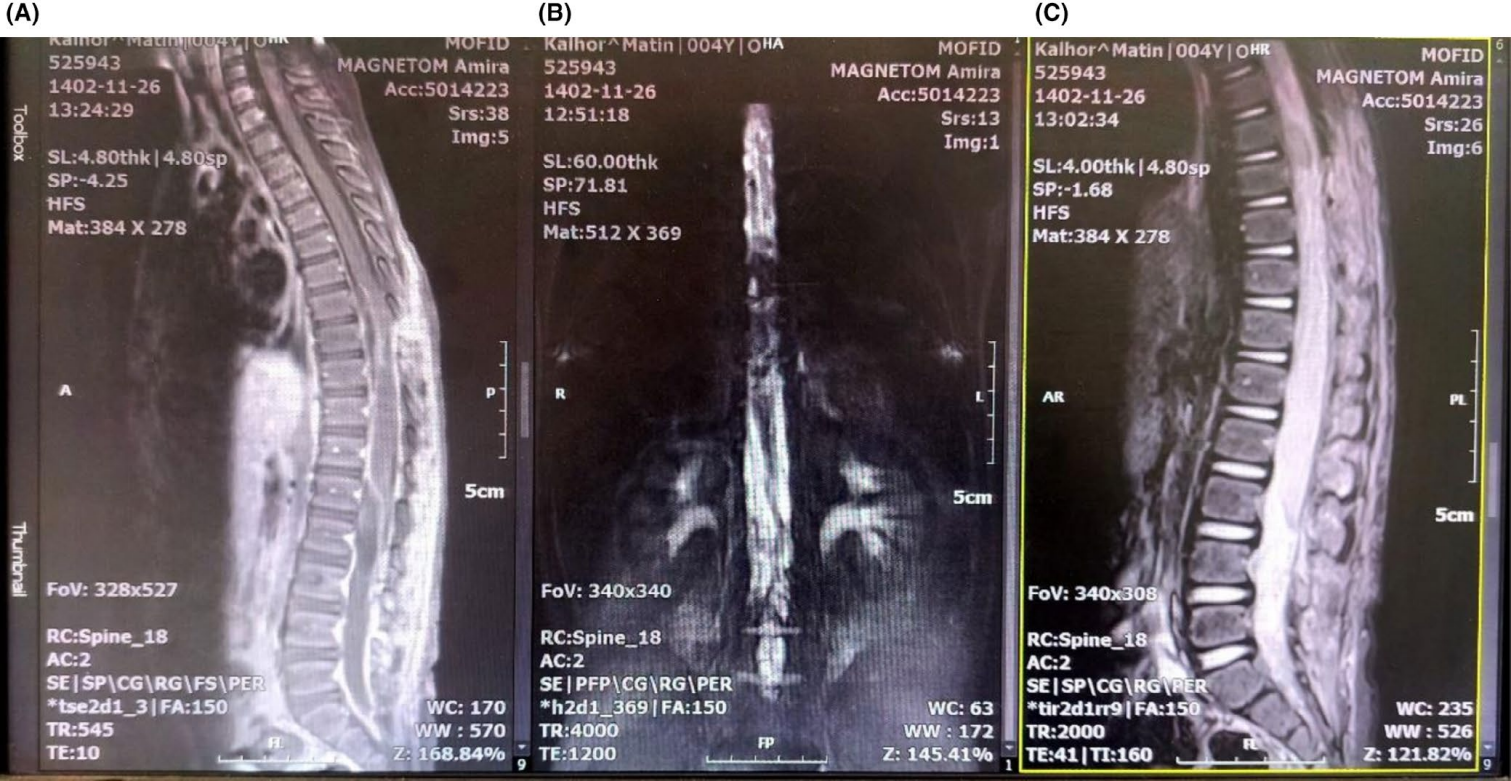
Early postoperative thoracolumbar imaging. (A) T1‐weighted MRI with gadolinium injection (left), (B) Myelogram (middle), (C) T2‐weighted MRI with gadolinium injection (right). They depict no residual hydatid cysts.

The patient was scheduled to undergo surgery with osteoplastic laminotomy of T10 to S2. The dura opened in the midline, and there were multiple cysts that were totally located in the intradural extramedullary space. The cysts had displaced the cord posteriorly (Figure 2). Cysts were pushed to the outside by saline irrigation, and all of them were resected without any residual cyst. Additionally, these cysts were assessed by an expert pathologist during the operation, which confirmed the diagnosis of hydatid cyst. Early postoperative thoracolumbar MRI showed no residual cysts (Figure 3). Medical treatment with albendazole (15 mg/kg/day orally in two doses for 6 months) and praziquantel (40 mg/kg/day orally in two doses for 4 weeks) was initiated. He experienced an unremarkable postoperative course. His lower limb muscle strength and urinary incontinence recovered after few weeks.

**FIGURE 4:**
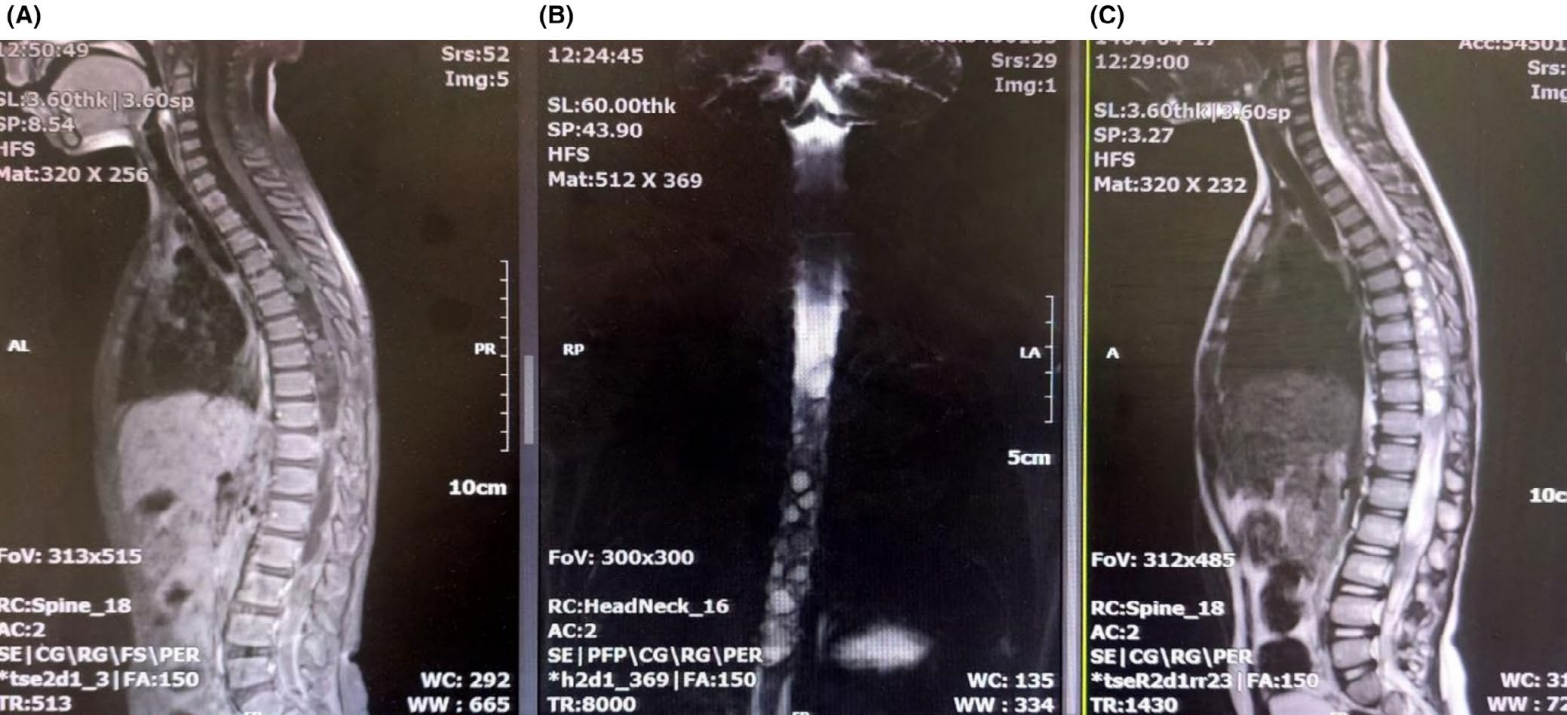
Postoperative thoracolumbar imaging after 10 months. (A) T1‐weighted MRI with gadolinium injection (left), (B) Myelogram (middle), (C) T2‐weighted MRI with gadolinium injection (right). Figures show recurrence of intradural multiple hydatid cysts with expansion to upper segments of thoracic spine.

At a 10‐month follow‐up, the thoracolumbar MRI showed recurrence of intradural multi‐hydatid cysts with expansion to the upper segments of the thoracic spine (Figure 4). However, the patient did not complain of exacerbating his symptoms.

## 5 Discussion

Hydatid disease of the spinal cord constitutes up to 1% of cases of hydatidosis. These cysts may range in size from the diameter of a pinhead to several centimeters. Interestingly, some of them can extend over a span of up to several vertebrae [13, 14]. As echinococcus reaches the spine through portovertebral venous shunts, hydatid cysts are mostly located posteriorly or posterolaterally to the spinal cord [12]. Hydatid cysts of the spinal cord may secondarily develop from origins in the lungs and pelvis or primarily from the spine. Unlike secondary epidural hydatid cysts, the occurrence of primary epidural hydatidosis without evident spinal involvement on radiological examination is extremely rare [11]. Our case was an intradural hydatid cyst, which is also very uncommon. Table 1 summarizes details of cases with intradural hydatid cysts reported in the literature.

**TABLE 1:** Details of cases with intradural hydatid cysts in the literature.

First author	Age (years), sex	Clinical manifestations	MRI findings	Finding during surgery	Outcome
Arif et al. [15]	9, male	Leg weakness	An intradural, extramedullary cystic lesion was seen extending from L1 to L4	An intradural, extramedullary cystic lesion from L1 to L4 spine measuring 9.8 × 9 × 1.8 cm	Improved
Zhang et al. [16]	17, male	Lumbar pain and leg weakness	A cystic lesion on T8 hypointense on T1‐weighted images and hyperintense on T2‐weighted	Whitish, pearl‐like, semitranslucent, cystic material	Improved
Işlekel et al. [17]	19, male	Back pain and neurologic claudication	Three round radiolucent lesions, 2–3 cm in diameter, one at the L3 and two at the L1 level	Multiple cysts measuring 5–30 mm in diameter were found floating among roots	Improved
Kaen et al. [18]	59, male	Lumbar pain and leg weakness	T12‐S1 Multiple cysts	Intradural multiple cysts	Recurrence
Kalkan et al. [19]	8, male	Back pain and difficulty in walking	A lobulated 3 × 1 cm cystic lesion with a regular contour. Intradural extramedullary at the T7‐T8 level	An intradural extramedullary cystic lesion	Improved
Kahilogullari et al. [20]	32, female	Lumbar pain and leg weakness	A 5 × 2 × 2 cm cystic lesion that was located intradurally at the L5‐S2 level	Two intradural cysts were removed with their capsules	Improved
Lotfinia et al. [21]	52, male	Back pain	6 × 9 × 2 cm intradural cystic lesion	Specimen measured 6 × 9 × 2 cm and was described as a whitish, pearl‐like, semi translucent	Improved
Güneçs et al. [22]	14, male	Lumbar pain and leg weakness	A large, multiloculated cystic lesion extending from L3‐S2 level, a lobulated 3 × 2 cm cystic lesion with a regular contourat the T5 level	A lot of pearly white hydatid cysts located intradural extramedullary	Improved
Lakhdar et al. [23]	22, male 5, male 10, female	Lumbar pain and leg weakness Cauda aquina syndrome Progressive weakness	A lobulated lesion measuring 22 × 15 mm in the intradural extramedullary at the T11 level An extramedullary mass lesion extending from T12 to the L5 level A cyst lesion extending from L2 to S1	Multiple unruptured intradural extra medullary hydatid cysts Multiple ruptured and unruptured vesicles A large irregular cystic mass in the thecal sac	Improved Complete recovery of his neurological deficit, except for some element of urinary stress incontinence Complete recovery
Akhan et al. [24]	6, male	Progressive leg weakness	An intradural, extramedullary mass between the levels T9 to T11	The dimensions of the cyst were 3 × 2 × 1 cm	Unspecified
Shukla et al. [25]	5, male	Progressive leg weakness	Well‐defined intradural cystic lesions from L4 to sacral region	An intradural cystic lesion	Improved
Şenol et al. [13]	38, female	Pain on her left arm and numbness on the left side of her body	a cervical intramedullary non‐contrast enhancing cystic lesion (1.2 × 0.7 cm) which was hypointense on T1 and hyperintense on T2 A paraspinal cystic lesion (2.5 × 1 cm) at the level of 5th and 6th thoracic vertebrae with liver cysts	Because of its high risks, neurosurgeons avoided surgery for intramedullary cyst and decided to follow the patient once a year regularly	Her clinical symptoms, such as, pain on her left arm and numbness on the left side of her body gradually got better
Hmamouche et al. [26]	72, female	Progressive leg weakness	Intradural process extending from L2 to S5, a low intensity on T1 weighted high‐intensity on T2 weighted	Multiple vesicles were found scattered among the roots, with no adhesion to meninges or nervous structures	Improved
Pushparaj et al. [10]	40, female	Progressive leg weakness	An intradural cystic lesion	A small bunch of pearly white cystic masses	Improved
Özmen et al. [27]	48, male	Numbness in the right leg and a medical history of Behçet's disease	An extramedullary mass lesion in the spinal canal, at T1 Level	Mass was located intradurally and extramedullary	Improved
Bettaieb et al. [28]	4, male 8, male 8, male 4, male 4, male 8, male 4, male	Paraplegia Paraplegia Paraplegia Paraplegia Paraplegia Paraplegia Paraplegia	Dorsal high Dorsal high Dorsal medium Dorsal Dorsal Dorsal Dorsal	Unspecified	Improved Improved Improved Improved Improved Improved Improved
Turan Süslü et al. [29]	34, male	Progressive back pain	T10‐T11 intradural multi‐cystic lesion	Yellowish white, solid, multi‐lobulated lesion	Improved
Midyat et al. [30]	13, female	Progressive leg weakness	T10‐T11 intradural multi‐cystic lesion	A solid, multi‐lobulated lesion	Improved
Secer et al. [31]	34, male	Progressive leg weakness	T11‐T12 intradural lesion	An intradural cystic lesion	Improved
Shukla et al. [32].	8, male	Lumbar pain and leg weakness	T12‐L2, intradural lesion	intradural transparent cystic lesion	Improved
Kalkan et al. [19]	8, male	Progressive leg weakness	T7‐T8, intradural lesion	An intradural cystic lesion	Improved
Erdogmus et al. [33]	43, female	Numbness in lower extremities for two years	T12‐L5 a multi‐septated intradural‐extramedullary cystic lesion	Multi‐septated intradural‐extramedullary cystic lesion	Improved
Hilmani et al. [34]	25, female	Cauda equina syndrome	An intradural process extending from L3 to L5, a low‐intensity signal on T1 weighted	Multiple vesicles were found scattered among the roots	Improved
Onbas et al. [35]	48, male	Progressive leg weakness	C7‐T1	Unspecified	Recurrence
Baurand et al. [36]	35, female	Paraplegia	T6	An intradural cystic lesion	Improved
Chat et al. [37]	13, female	Paraplegia	T5‐T11, L4‐L5	An intradural cystic lesion	Improved
Islekel et al. [17]	19, male	Paraplegia	L2_L4	Multiple hydatid intradural cysts	Improved
Pamir et al. [8]	34, female	Paraplegia	L2	An intradural extramedullary cyst	No change
Sharma et al. [38]	16, female	Paraplegia	T6‐T10	An intradural extramedullary cyst	Improved
Sánchez et al. [39]	52, female	Paraplegia	L2	Two large and several small cysts	Improved
Chakir et al. [40]	18, male	Paraplegia	L1‐L2 intradural cyst	Intradural cystic lesion extending from L1 to L2	Improved
Bioxados [41]	4, female	Paraplegia	T5	Intradural extramedullary	Improved
Carrea et al. [42]	9, female	Paraplegia	T4‐T5	Intradural extramedullary	Improved
Karvounis et al. [43]	37, female	Siatica	L5‐S1	Intradural extramedullary	Improved
Medjek et al. [44]	21, female	Paraplegia	T12‐L1	Intradural extramedullary	Improved
Kabbaj‐El Kouhen et al. [45]	6, male	Paraplegia	L1‐L3	Intradural extramedullary	Improved

As hydatid disease is a multiorgan condition, it is required to examine the thorax and abdominopelvic cavity whenever a spinal hydatid cyst is diagnosed. Progressive enlargement of the cysts can destroy bones and disseminate epidurally, resulting in spinal compression and subsequent neurological symptoms [46, 47]. When the spine is affected by hydatid cysts, backache typically emerges as the primary presentation. Weakness of the lower extremities may develop in advanced cases. Other manifestations that may emerge include radiculopathy, myelopathy, and pathological fractures [48]. Disease may progress rapidly and require emergency surgical intervention, or it may have a slow course as in our patient [1].

There is no clear radiological appearance that definitively diagnoses spinal hydatid cysts. Plain radiographic examination may demonstrate nonspecific findings, such as osteolytic bony destruction and a soft tissue mass. Spinal CT scan indicates bone erosion and lesion extension with the utmost precision. In cases of spinal deformity, CT scans can also accurately reveal it. Calcification, which appears as a “double layer of arcuate calcification” can assist clinicians in diagnosing spinal hydatidosis. The most sensitive imaging modality for diagnosing spinal hydatid disease is MRI [1], which shows a well‐defined cystic mass inside the spinal canal. The cyst appears hypointense on the T1‐weighted image and hyperintense on the T2‐weighted image, respectively [11]. The wall of the cysts is regular and thin. They may exhibit similar or a somewhat lower signal compared with their inside. A slight enhancement may be observed following gadolinium injection, which indicates pericystic vessels. Arachnoid cyst, arachnoiditis, cystic tumor, tuberculosis, and cysticercosis are among the differential diagnoses of hydatic cyst [16]. Due to nonspecific clinical manifestations and imaging findings, it is challenging for clinicians to diagnose hydatidosis of the CNS. Nevertheless, in suspected cases serologic assessments can help diagnose the disease.

The treatment of echinococcosis is achieved with antiparasitic medications (e.g., albendazole and praziquantel) to eliminate the larvae and decrease the inflammatory process. Surgical intervention is also required in some cases to excise the cysts and prevent their recurrence. Minimally invasive techniques like stereotactic aspiration or endoscopic cyst removal can be utilized to drain cysts without the need for open surgery [7, 49]. For intradural hydatid cysts, surgery is the primary treatment method. It is essential to exercise caution during the procedure to avoid cyst rupture or tearing of the dura, as these complications can increase the risk of dissemination and recurrence. Long‐term follow‐up is important to monitor for any recurrence of the condition [1, 50].

## 6 Conclusion

Spinal cord involvement with hydatid cysts is an extremely rare form of echinococcosis that can lead to the compression of the spinal cord in pediatrics. Given its high incidence and devastating complications, clinicians should consider it as a differential diagnosis of spinal cord masses, particularly in endemic regions. Preoperative clinical suspicion and intraoperative judgment are of great importance for timely diagnosis and management of spinal hydatid disease, which can prevent devastating complications, such as cyst expansion and spinal cord damage. Furthermore, the surgical technique should be performed gently to prevent cyst rupture and dural tears. Due to the high recurrence rate, patients should be followed up for a long period of time.

## Author Contributions

**Afshar Abolhasani:** conceptualization, data curation, investigation, writing – original draft. **Abdolnaser Farzan:** conceptualization, data curation, supervision, writing – review and editing. **Amirreza Keyvanfar:** data curation, investigation, writing – review and editing. **Mahtab Khodabandelu:** data curation, investigation, writing – original draft.

## Funding

The authors have nothing to report.

## Ethics Statement

Due to regional regulations, case reports do not require ethical approval code.

## Consent

Written informed consent has been obtained from the parents of the patient.

## Conflicts of Interest

The authors declare no conflicts of interest.

## Data Availability

The data that support the findings of this study are available from the corresponding author upon reasonable request.
